# Supplementary material for: Estimation of Hg(II) in Soil Samples by Bioluminescent Bacterial Bioreporter E. coli ARL1, and the Effect of Humic Acids and Metal Ions on the Biosensor Performance
Source: Sensors (Basel). 2020 Jun 2;20(11):3138. doi: 10.3390/s20113138 (PMC7308967; doi:10.3390/s20113138)
Supplement: Supplementary file 1 [file sensors-20-03138-s001.pdf]

*Supplementary material*

# Estimation of Hg(II) in soil samples by bioluminescent bacterial bioreporter *E. coli* ARL1, effect of humic acids and metal ions on the biosensor performance

**Irena Brányiková [1], Simona Lucáková [1,2], Gabriela Kuncová [1,3], Josef Trögl [3,*], Václav Synek [3] , Jan Rohovec [4] and Tomáš Navrátil [4]**

a)

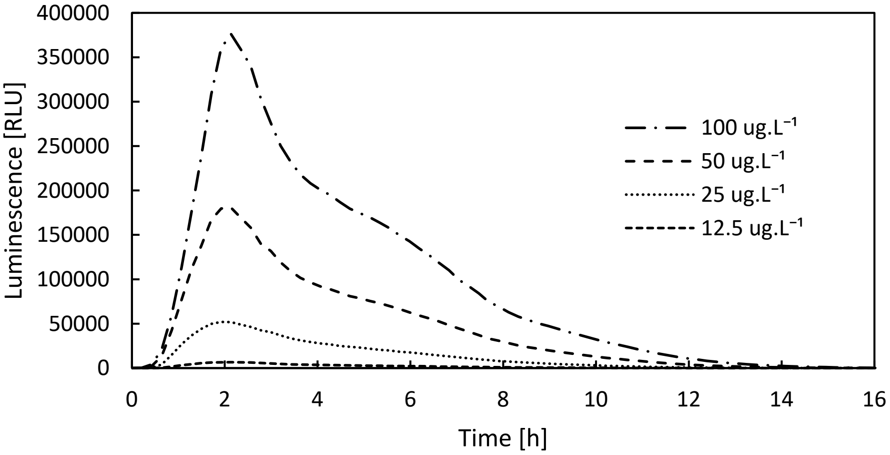


b)

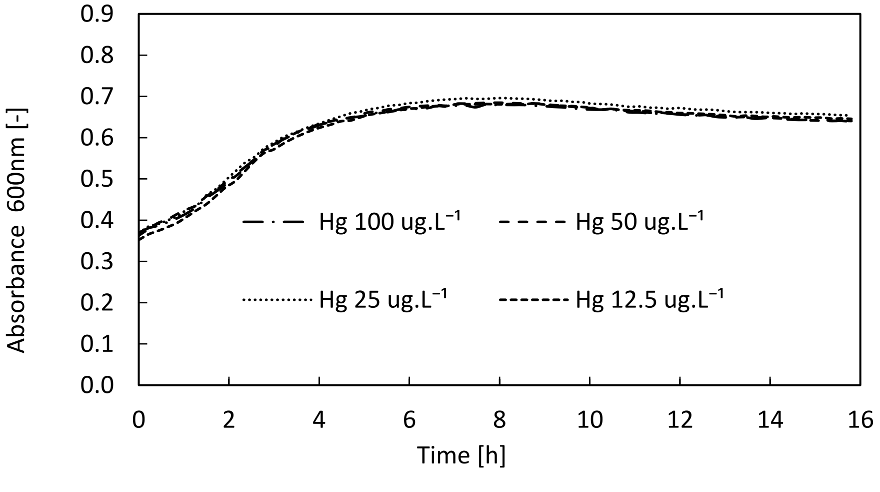


**Figure S1.** Sample bioluminescence response (**a**) and corresponding changes in the $OD_{600}$ during the measurement.
